# A case of oral cancer with delayed occipital lymph node metastasis: Case report

**DOI:** 10.1002/ccr3.3086

**Published:** 2020-07-30

**Authors:** Kisho Ono, Norie Yoshioka, Masanori Masui, Kyoichi Obata, Yuki Kunisada, Tatsuo Okui, Soichiro Ibaragi, Hotaka Kawai, Hitoshi Nagatsuka, Akira Sasaki

**Affiliations:** ^1^ Department of Oral and Maxillofacial Surgery Dentistry and Pharmaceutical Sciences Okayama University Graduate School of Medicine Okayama Japan; ^2^ Department of Oral Pathology and Medicine Dentistry and Pharmaceutical Sciences Okayama University Graduate School of Medicine Okayama Japan

**Keywords:** delayed metastasis, lymphatic regurgitation, neck dissection, occipital lymph node, oral cancer

## Abstract

Consideration of unexpected metastasis in patients who have undergone neck dissection with advanced tumors must be anticipated with careful follow‐up.

## Graphical abstract

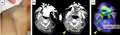

## 1 INTRODUCTION

Neck lymph node (LN) metastasis is common in patients with oral cancer (OC), but occipital LN metastasis in OC patients has not yet been reported. In this case report, we describe an OC patient with delayed metastasis to the occipital LN in the ipsilateral side.

Treatment and control of regional lymph node (LN) metastasis are an important prognostic factor in oral cancer (OC). The incidence of neck LN metastases in OC is 20%‐30%.^1^, ^2^, ^3^, ^4^, ^5^ In addition, the chance of developing a neck LN metastasis after initial treatment of OC varies from 12.4% to 62%.^6^, ^7^ The prognosis is poor in patients with neck LN metastasis of OC, and the survival rate varies depending on the degree of metastasis. The appearance of metastases to the neck LNs subtly differs depending on the subsite of primary lesion in OC, but most are often found in the neck LNs including the submandibular and the superior internal jugular region. However, to our knowledge, metastasis to the occipital LN in OC is very rare and has not been reported to date. In this paper, we report a case of OC with delayed metastasis to occipital LN in the ipsilateral side after neck dissection.

## 2 CASE REPORT

**FIGURE 1:**
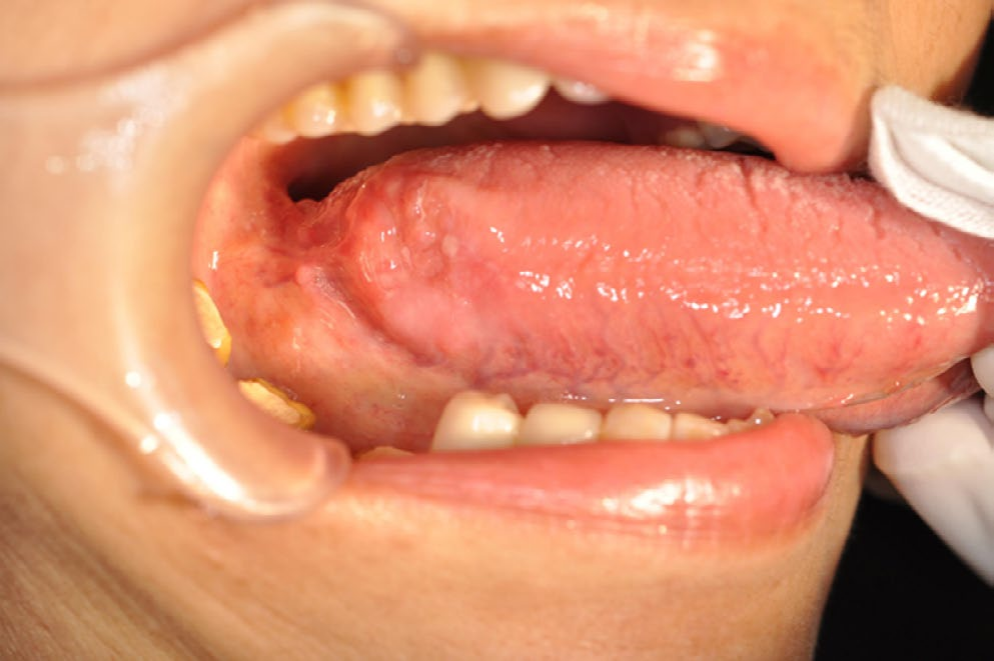
Intra‐oral findings at first physical examination. A tumor was located on the right lateral edge of the tongue

**FIGURE 2:**
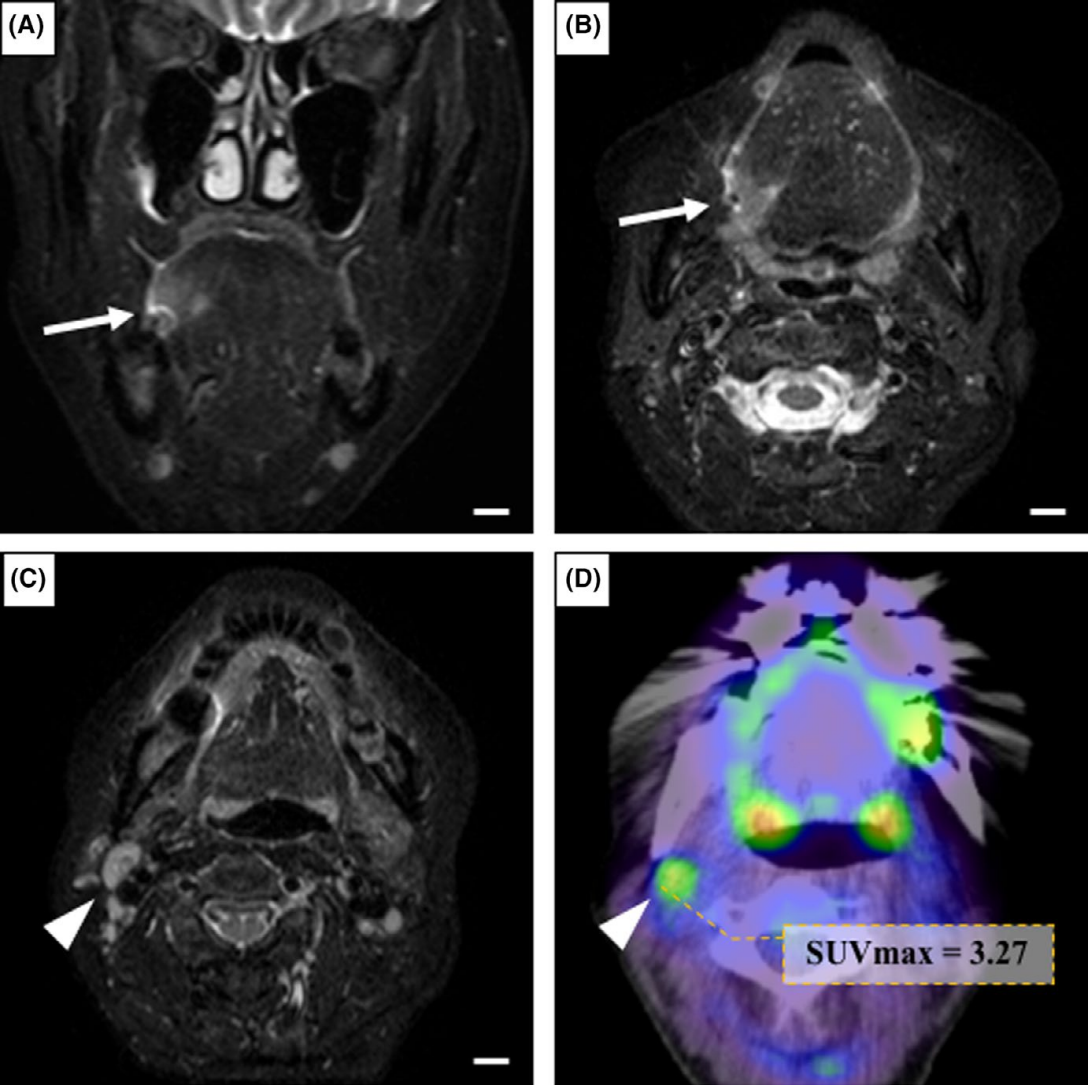
A, B: Axial (A) and coronal (B) fat‐suppressed T2‐weighted MR image showing the primary tumor (arrow) with inward extension on the right lateral edge of the tongue. Scale bar: 10 mm. C: Coronal fat‐suppressed T2‐weighted MR image showing swelling of LN (arrowhead) in the right superior internal jugular region. Scale bar: 10 mm. D: PET‐CT showing abnormal accumulation of FDG at the right superior internal jugular region (SUVmax = 3.27)

A 62‐year‐old woman was referred by her dentist to our department in May 2013 with a suspected malignancy of the right tongue. The patient did not complain of pain, but uncomfortable stiffness of the tongue. She had no history of trauma, disease, surgery, or smoking, and only a small amount of daily drinking. Physical examination showed an ulcerative lesion measuring 14 × 10 mm with induration was recognized on the right lateral edge of the posterior tongue at the first visit (Figure 1). The submandibular LNs of 8 mm diameter were palpable, mobile, and not tender. The results of cytology of the lesion in the oral cavity showed Class IV. Ultrasonography showed the presence of a tumor with a thickness of about 8 mm inward at the lesion site. Head and neck contrast‐enhanced computed tomography (CE‐CT) imaging strengthened the clinical suspicion of an OC of the right lateral edge of the tongue. Magnetic resonance (MR) imaging revealed the primary tumor extended into the right tongue region, inward with thickness of 10 mm or more, and LNs enlarged at the right superior internal jugular region (Figure 2A‐C). Correspondingly, positron emission tomography (PET)‐CT showed abnormal accumulation of FDG in the right lateral edge of the tongue (SUVmax = 6.98) and LNs at the right superior internal jugular region (SUVmax = 3.27), and no abnormal accumulation of FDG in other organs (Figure 2D). As a part of the staging and planning procedures, biopsy was performed, and the result was confirmed to be squamous cell carcinoma (SCC). Accordingly, the patient was clinically diagnosed with T2N1M0 stage III in the TNM classification of the 7th edition at that time.

**FIGURE 3:**
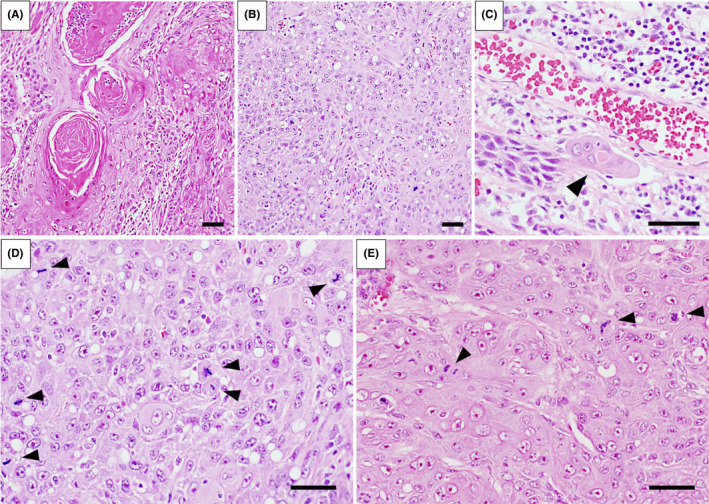
Hematoxylin and eosin staining showing pathological features of resected specimen at initial surgery. Scale bar: 50 μm. A, B: The primary tumor had predominantly moderately differentiated regions (A) with some poorly differentiated regions (B). C: Vascular invasion findings of tumor cells (arrow head) at primary tumor. D, E: Cell division findings of tumor cells (arrow heads) at primary tumor lesion (D) and neck LN metastatic lesion (E)

In June 2013, we performed partial tongue resection including tumor lesion and modified radical neck dissection (type III; preserving sternocleidomastoid muscle, internal jugular vein, and accessory nerves) at the right side. The histopathological examination of the resected tongue specimen showed a predominantly moderately differentiated SCC with some poorly differentiated regions (Figure 3A, B) and findings of vascular invasion of tumor cells (Figure 3C), and the resected neck LNs specimen showed only one nodal metastasis at level IIa LN (pT2 pN1 (1/50) Ly1 V1 Pn0 G2 > G3, local R0). Interestingly, many cell divisions were observed in both resected specimens depending on the site, which were 16 per 10 high‐power field (HPF) of primary tumor lesion and 13 per 10 HPF of neck LN metastatic lesion (Figure 3D, E). Complete tumor disappearance resulted from surgery, and we decided that no additional treatment was needed as histology confirmed that there were negative resection specimen margin and no extranodal extension (ENE).

**FIGURE 4:**
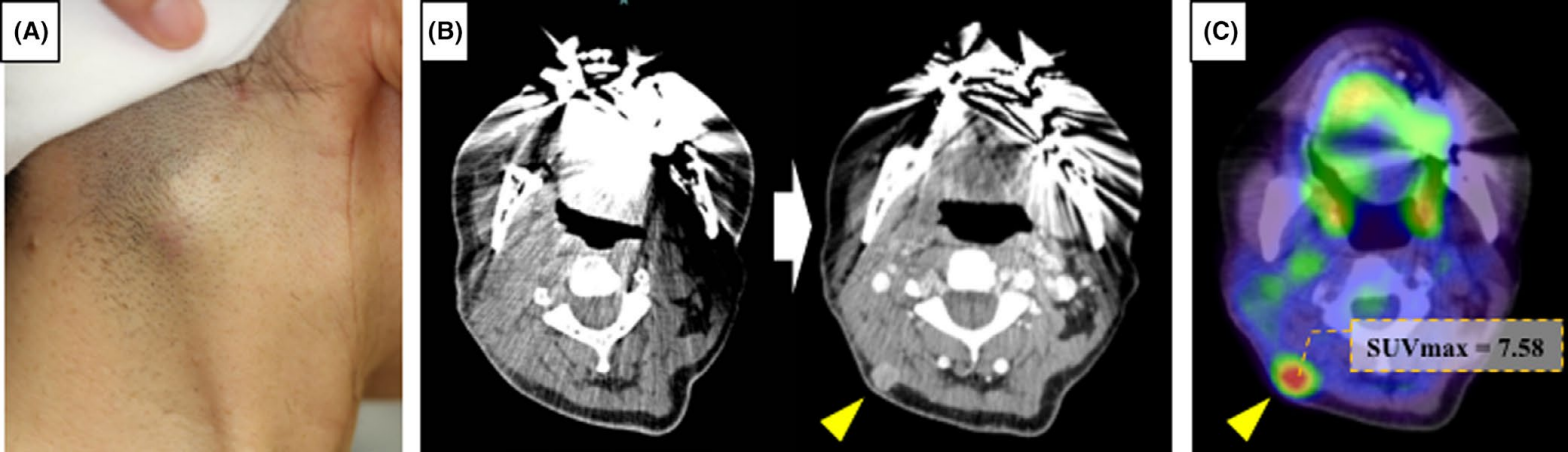
A: Right posterior neck findings at 3 mo after initial surgery. A subcutaneous mass enlargement was palpable. B: CE‐CT image showing a swelling of the right occipital LN (arrow head) suspected of metastasis. CT images 2 (left) and 3 mo (right) after initial surgery. C: PET‐CT showing abnormal accumulation of FDG at the right occipital region (SUVmax = 7.58)

However, at the time of follow‐up in September 2013, a subcutaneous mass enlargement of the right posterior neck on the ipsilateral side was observed (Figure 4A). Ultrasonography revealed a 10 mm bulge near the trapezius muscle on the right posterior neck. Furthermore, CT imaging showed that the occipital LN had a tendency of rapid swelling and contrast enhancing compared with which was taken a month ago (Figure 4B). Correspondingly, PET‐CT showed abnormal accumulation of FDG (SUVmax = 7.58) in the ipsilateral posterior neck (Figure 4C). As a side note, physical examination and these image examinations did not find local recurrence of the tongue or metastasis to the contralateral neck LN.

**FIGURE 5:**
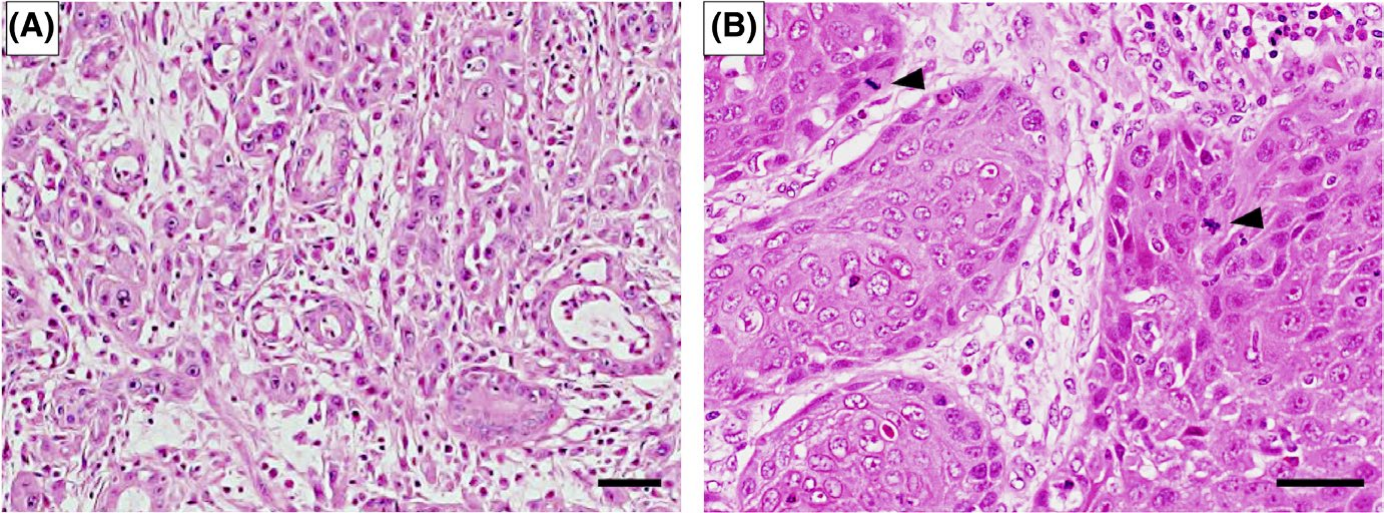
Hematoxylin and eosin staining showing pathological features of resected occipital LN. Scale bar: 50 μm. (A) The delayed tumor had predominantly moderately differentiated lesion. (B) Cell division findings of tumor cells (arrow heads) at delayed tumor lesion

Suspected delayed LN metastasis was considered and lymphadenectomy was performed for the purpose of pathological examination. The histopathological examination of the resected specimen showed presence of proliferating tumor cells, forming tumor nests. Tumor tissue was moderately differentiated SCC, accompanied by many cell divisions (11 per 10 HPF), and the LN‐specific structure disappeared (Figure 5). Based on the findings above, we considered that additional treatment was necessary, and the patient was treated with bioradiation (BRT), cetuximab (initial dose of 400 mg/m^2^ with subsequent weekly doses of 250 mg/m^2^ intravenously, total 8 courses) combination radiotherapy (2 Gy/day, total dose of 60 Gy). Subsequentially, we have been conducting regular follow‐up, but thus far it has been 7 years with no sign of local recurrence, other LNs metastasis, and distant metastasis.

## 3 DISCUSSION

Oral cancer is an aggressive disease that mainly metastasizes to neck LNs. The extent of neck area invasion directly affects the prognosis of the patient, regardless of the number, the size of metastatic LNs, and the presence of ENE.^8^ Akhter M et al^9^ found that the prognosis of OC patients with neck LN metastasis was worse than those of without LN metastasis, and metastases to the LN nodes were more likely to develop distant metastases. The most common pattern of neck LN metastasis of OC is level I or II LNs.^10^, ^11^ On the other hand, the rate of metastasis to level V LNs was low, and Lim et al showed only 5% (5 of 93) of OC and oropharyngeal cancer with clinically N positive (cN+) had positive of level V LNs, and all of which cases also had positive of other level LNs.^12^ Parikh et al also reported that 0% (0 of 99) of cN1 with palpable level Ib LNs, 8% (1 of 13) of cN1 with palpable level II/ III LNs, and 16% (8 of 49) of cN2 were positive of level V LNs.^13^ In addition, there are many cases in which latent metastases are found in cN0 cases with no clinically recognized LN metastasis. Li et al^14^ and Parikh et al^13^ reported that about 20% of cN0 had potentially latent LN metastasis. However, all of them were negative of level V LNs. Incidentally, studies of LN metastasis in the head and neck region, including oral cavity, did not reveal a lymphatic drainage route to the occipital LNs.

Occipital LNs are located at level Xb LN in “the guidelines of the lymph node levels modified from Robbins.^15^” The occipital LNs are located at the junction of the calvaria and the apex, and divided into three places, such as (a) suprafascial/superficial, (b) subfascial and (c) submuscular/ subsplenius, from the surface layer to the deep part according to the anatomical relationship with the fascia and muscle. The import lymph vessels of the occipital LN include the scalp of the occipital region, the skin of the apex, the deep layer of the occipital neck, and the export lymph vessels which mainly flow into the upper part of the accessory nerve LNs.^16^ Maeda et al reported that 67% (4 of 6) of the occipital skin cancers spreading to the occipital LNs showed metastasis to level V LNs,^17^ and this result supported that the efferent lymphatic vessels of the occipital LNs are accessory nerve LNs. Furthermore, it is known that a part of occipital lymph vessels also drains into the jugulodigastric LN located in neck node level IIa.^18^ The jugulodigastric LNs form part of the upper internal jugular LNs located in the carotid trigone. Especially in the head and neck region, tumors of the base of the tongue, the pharynx, and the tonsils are often metastasized to jugulodigastric LNs.^19^

Lymph node metastasis shows a metastatic pattern along the lymph flow from the primary lesion, and the level at which metastasis frequently occurs has been clarified depending on the primary site. Therefore, preservation of LN groups with a rare metastasis frequency does not impair treatment results. Accordingly, neck dissection is performed in advanced cases, but antegrade lymphatic flow may be blocked by neck dissection itself or obstruction of adjacent lymph vessels associated with tumor progression. As a result, lymphatic regurgitation may occur, and residual tumor cells may unexpectedly disseminate by a route that ignores the original lymphatic flow.^20^, ^21^, ^22^ Yang et al hypothesized that extensive neck LN metastasis, particularly meeting the condition of N3, was a risk factor for lymphatic regurgitation to the occipital LN metastasis.^23^ However, in the case we experienced, the pathological results of the LNs dissected in the initial surgery showed metastasis was found only in level IIa LN, but delayed metastasis to the occipital LN was found at 3 months postsurgery. Li et al showed that extensive obstruction of internal jugular vein nodes caused by neck LN metastasis of nasopharyngeal cancer may occur lymphatic regurgitation to occipital LN^24^. In this case, we cannot determine the cause of occipital LN metastasis, but it was suggested that the following two points may be involved: (a) changes in lymph flow due to neck dissection and (b) obstruction of internal jugular LNs due to tumor progression.

For LN metastasis, it is important to confirm lymphadenopathy, induration, and mobility by palpation. Besides that, imaging test is often useful for diagnosing metastatic LNs, and occipital LN metastases are visualized as a superficial mass located at the cranial and superficial of posterior neck.^25^ CT may confirm metastases regardless of size if central necrosis is present. CE‐CT shows LN central necrosis as a central area of low attenuation surrounded by an irregular rim‐enhancing tissue.^26^, ^27^ On the other hand, pathologically, the LN structure may have already been impacted at the time when the metastasis was clinically revealed as in this case; thus, it was difficult to prove whether it was true LN metastasis. However, in this case, we considered the delayed tumor was occipital LN metastasis from the primary tongue tumor by the following reasons: Tumor was found in the position of occipital LN by CE‐CT early postoperation, the histological type was moderately differentiated SCC with many cell divisions consistent with the primary tumor, and a whole‐body search using PET‐CT revealed no malignant tumor in another organs.

The TNM classification is the international standard for the progression of malignant tumors advocated by the American Joint Committee of Cancer (AJCC) and the Union for International Cancer Control (UICC). Although the definition of T classification (T1 to T3) of OC had not been revised for almost 30 years, the concept of depth of invasion (DOI) of the primary lesion was added in the revised 8th edition of the AJCC/UICC staging system published in 2017. Specifically, in addition to the concept of the maximum tumor diameter of the 7th edition, the T classification of the 8th edition added the criteria of being classified as T2 or more when the DOI exceeds 5 mm and T3 when the DOI exceeds 10 mm. We initially diagnosed T2N1M0 based on the size of the primary tumor and the presence or absence of neck LN metastasis. However, according to the classification of the 8th edition, it should be classified as T3 because the tumor depth of ≥10 mm was recognized on the MR image (Figure 2). There have been reports that tumor depth is a risk factor for delayed neck LN metastasis.^28^, ^29^ The International Consortium for Outcome Research (ICOR) in Head and Neck Cancer conducted an international large‐scale retrospective study of 3149 OC cases in 22 years at 11 centers. As a result, it was proved that the DOI was a more important factor in poor prognosis rather than the size of the tumor itself.^30^ In our case as well, the depth of the tumor, which was not evaluated at that time, may have led to a poor postoperative outcome.

Metastasis to the occipital LNs is very rare in the first place and can occasionally occur in cases such as malignant tumors of the occipital skin and skin appendages, malignant tumors of the ear canal, and melanoma of the head and neck. Other rare cases of occipital LN metastasis have been reported in sweat gland tumors,^31^ lung cancer,^32^ and thyroid cancer.^33^, ^34^ In head and neck cancer, it was reported that there was no risk of metastasis to the occipital LNs, except in the case of skin,^16^ but as mentioned above, there are reports of metastasis to the occipital LNs in nasopharyngeal cancer.^23^, ^24^ To our knowledge, our case is the first report of occipital LN metastasis arising from oral cavity. Various treatments have been applied to occipital LN metastases of each cancer type. Lin et al^33^ performed dissection of the occipital LN with resection of the primary lesion in a case of papillary thyroid cancer. No further treatment was given, and the patient died 17 months later due to brain metastases. Sheth et al^31^ performed radiation therapy as an additional treatment after the surgery in a case of eccrine mucinous cancer and achieved disease‐free survival of 4 years. After that, recurrence and metastasis were repeatedly observed, but a long‐term survival course was achieved by performing tumor resection and radiotherapy each time. Yang et al^23^ performed chemoradiation therapy combined with cisplatin and 5‐fluorouracil as an additional treatment after tumor resection in a cases of nasopharyngeal cancer. After that, distant metastases were found in the sternum, left iliac, bilateral breast LNs, and spleen, but a long‐term survival course was achieved without any recurrence in the primary region and related areas. Normally, in head and neck cancer, metastases are rarely found in the LNs of occipital, parotid, and facial, which are outside of the normal dissection area. Furthermore, metastases in these areas have been reported to have a poorer prognosis than metastases to neck LNs (level I–V) within the dissection area.^35^, ^36^, ^37^ We performed BRT combined with cetuximab as an additional treatment after excision of metastatic LN and achieved good progress with a survival time of 7 years or more. Although there are some risks of recurrence and/or distant metastasis, these cases mentioned above including our case suggest that comprehensive methods and multidisciplinary management including surgery, chemotherapy, and radiation therapy used for common cancer metastases may be important even to unexpected metastasis.

## 4 CONCLUSION

Metastasis to regional LNs for OC, such as occipital LN, is very rare, but we should consider the changes of lymph flow in the patients who underwent neck dissection with advanced tumors, and follow up them consistently and carefully.

## CONFLICTS OF INTEREST

The authors declare that there are no conflicts of interest regarding the publication of this paper.

## AUTHOR CONTRIBUTIONS

KOn and YO: wrote the manuscript. YO, MM, SI, and AS: engaged in the treatment of the patient. KOn, YO, MM, KOb, YK, and TO: discussed the significance of the case. HK and HN: provided the pathology images and detailed findings for the case. All authors reviewed and finalized the manuscript.

## CONSENT

Written consent from the patient has been obtained.
